# Early embryonic lethality in complex I associated* p.L104P* *Nubpl* mutant mice

**DOI:** 10.1186/s13023-022-02446-y

**Published:** 2022-10-24

**Authors:** Cheng Cheng, James Cleak, Lan Weiss, Heather Cater, Michelle Stewart, Sara Wells, Rod Carlo Columbres, Alyaa Shmara, C. Alejandra Morato Torres, Faria Zafar, Birgitt Schüle, Jonathan Neumann, Eli Hatchwell, Virginia Kimonis

**Affiliations:** 1 Division of Genetics and Genomic Medicine, Department of Pediatrics, University of California, Irvine, CA USA; 2 Mary Lyon Centre, MRC Harwell Institute, Harwell Campus, Oxfordshire, OX11 0RD UK; 3 Department of Pathology, Stanford University School of Medicine, Stanford, CA USA; 4 Transgenic Mouse Facility, University of California, Irvine, CA USA; 5 Population Bio UK, Inc, Begbroke Science Park, Begbroke, UK; 6 Department of Neurology, University of California, Irvine, CA USA; 7 Department of Pathology, University of California, Irvine, CA USA; 8 Department of Environmental Medicine, University of California, Irvine, CA USA; 9 Children’s Hospital of Orange County, Orange, CA USA

**Keywords:** Complex I deficiency, Mitochondria, Parkinson’s disease, NUBPL, Mouse model

## Abstract

**Background:**

Variants in the mitochondrial complex I assembly factor, NUBPL are associated with a rare cause of complex I deficiency mitochondrial disease. Patients affected by complex I deficiency harboring homozygous *NUBPL* variants typically have neurological problems including seizures, intellectual disability, and ataxia associated with cerebellar hypoplasia. Thus far only 19 cases have been reported worldwide, and no treatment is available for this rare disease.

**Methods:**

To investigate the pathogenesis of NUBPL-associated complex I deficiency, and for translational studies, we generated a knock-in mouse harboring a patient-specific variant *Nubpl* c.311T>C; p. L104P reported in three families.

**Results:**

Similar to *Nubpl* global knockout mice, the *Nubpl* p. L104P homozygous mice are lethal at embryonic day E10.5, suggesting that the *Nubpl* p. L104P variant is likely a hypomorph allele. Given the recent link between Parkinson’s disease and loss-of-function *NUBPL* variants, we also explored aging-related behaviors and immunocytochemical changes in *Nubpl* hemizygous mice and did not find significant behavioral and pathological changes for alpha-synuclein and oxidative stress markers .

**Conclusion:**

Our data suggest that homozygotes with *Nubpl* variants, similar to the null mice, are lethal, and heterozygotes are phenotypically and neuropathologically normal. We propose that a tissue-specific knockout strategy is required to establish a mouse model of *Nubpl*-associated complex I deficiency disorder for future mechanistic and translational studies.

## Introduction

Mitochondrial Complex I deficiency is the third most common mitochondrial disorder in the population. In humans, complex I is composed of 44 different subunits either encoded by nuclear or mitochondrial DNA [1]. More than 16 genes are implicated in mitochondrial complex I assembly [1], and pathogenic variants in subunits or assembly factors have been implicated in the complex I disorders. Nucleotide-binding protein-like (NUBPL) plays a critical role in complex I assembly. Recessive variants in the *NUBPL* gene are causative for a rare mitochondrial complex I deficiency disorder. Previous studies have investigated the pathogenicity of *NUBPL* mutations in complex I assembly in the yeast model. In particular, a yeast mimic of the patient-specific variant, *NUBPL* p.L102P significantly reduced the NUBPL protein stability and complex I assembly, suggesting that this variant leads to significant loss of NUBPL function, thus disrupting complex I assembly [2].

The NUBPL gene was first reported as a cause of mitochondrial complex I deficiency (MIM 613621, 618242) in 2010 in a whole exome sequencing (WES) study of over 100 patients with clinical and biochemical evidence of complex I deficiency [3]. Six additional patients were identified from a magnetic resonance imaging (MRI) database of more than 3000 subjects with unclassified diffuse leukodystrophy involving the cerebellar cortex, periventricular deep and subcortical white matter and corpus callosum with some cystic changes [4]. All patients from this study had c.815-27T>C in cis with c.166G>A plus a second deleterious NUBPL variant in trans. Three more families were reported to have NUBPL disease [2–8].

We recently reported four new patients with compound heterozygous variants in the *NUBPL* gene, among whom two families had the *NUBPL* p. L104P variant [9]. The affected individuals presented with ataxia, cerebellum hypoplasia, global developmental delay, and one patient had a Leigh-like phenotype variant [9]. Literature review identified one additional family who was compound heterozygous with the p. L104P *NUBPL* variant [9], the other allele being the branch-site c.815-27T>C variant. Studies have suggested that this splicing site variant c. 815-27T>C can lead to incidences of exon 10 skipping and intron inclusion, thus reducing the normal NUBPL mRNA level [2, 9]. Notably, the c. 815-27T>C occurs at a frequency of 1.2% in the European population, which suggests that this variant is mild. The more severe p. L104P variant has not been well-characterized in the population, and its function in a rodent model has yet to be determined.

To understand the role of *NUBPL* variants in disease pathogenesis, we generated the *Nubpl* global knockout mice, and the patient-specific *Nubpl* p. L104P knock-in mice using CRISPR/Cas9 technology. Consistent with the prior studies [10], we found early embryonic lethality in both homozygous models, indicating that the *NUBPL* p. L104P variant is deleterious and behaves similar to the null allele.

A recent report suggests that loss of function in *NUBPL* may increase the susceptibility of Parkinson’s disease (PD) in adulthood [11]. Since the aging phenotype of the heterozygote mice had not been studied, we examined the PD-associated behaviors in aging *Nubpl* heterozygous mice to investigate a possible link between NUBPL deficiency and PD.

## Methods

### Generation of the heterozygous* Nubpl*^L104P+/−^ knock-in mice using CRISPR/Cas9 technology

*Nubpl*^*104P+/-*^ mice were generated using CRISPR/Cas9. C57BL6N/J pronuclear staged zygotes were injected with gRNAs (2uM each), Cas9 protein (3uM IDT), and p. L104P ssODN repair template. The resulting offspring were genotyped and sequenced. Founder mice with the p. L104P variant were mated with C57BL6N/J. F1 generation p. L104P mice were sequenced to confirm mutant sequence (F primer: 5′ AGCTATCCCAGGCAATTTATACTT 3′; R primer: 5′ GCCTGGAAAGTGAACAGTAGGT 3′). Subsequent genotyping used Taqman probes (Nubpl WT probe: CTTGTTAGATGTGGATGT; Nubpl mutant probe: CTTGCCAGACGTCGAC). The mice have been backcrossed for 10 generations to remove the contaminating background.

### Generation of the hemizygous* Nubpl* knock out mice

*Nubpl* Knockout first (reporter-tagged insertion with conditional potential) ES cells and mice (allele name:* Nubpl*^tm1a(EUCOMM)WTSI)^, MGI:4363128) were generated by the International Mouse Knockout Consortium (https://www.mousephenotype.org/), via a mouse embryonic stem (ES) cell-targeting approach using C57BL/6NTac ES cells, such that exon 4, a critical exon, has the KO first cassette inserted.

### Embryo harvesting of the homozygous Nubpl knockout and* Nubpl*^*L104P/L104P*^ mice

Heterozygous mice were mated and embryos were timed based on detection of a vaginal plug. The following morning was considered to be 0.5 dpc. At 9.5–11.5 dpc pregnant females were sacrificed by cervical dislocation and the uterine horns dissected out. Individual embryos were removed from the placentae and placed in individual wells of a Corning Costar 12-well plate and imaged. Images were taken using a ProgRes Speed XT^core^5 Jenoptik camera attached to a Leica M165-C stereomicroscope with a Leica Objective Planapo 0.63x lens and integrated LED ring light. Genotyping was performed at Transnetyx with self-designed primer sets.

### Behavioral tests of hemizygous Nubpl knockout mice

The hemizygous *Nubpl* mice were subject to a battery of tests (Table 1) as previously described [6] at 14 months to identify PD-like phenotypes. Behavioral tests were conducted at MRC Mouse Genetics Research Institute. Terminal behavioral analyses were based on The Adult Phenotype Pipeline from the International Mouse Phenotyping Consortium (IMPC) (Table 1) (https://www.mousephenotype.org/impress/index).

**Table 1:** Evaluation of aging *Nubpl*+/− mice

Motor	Aging parameters	Vision	Emotionality
Open field	Tremor	Visual placing	Aggression
Grip strength	Vocalization	Lens mass	Startle response
Contact righting	Fat mass	Overall health of cornea, eye, eyelid, iris, pupil, retina, eye vasculature	
Gait	Bone mineral content and density		
Head bobbing			

### mRNA analysis to confirm expression

mRNA was isolated from heterozygous brain and quadricep muscle. cDNA was made from mRNA using random priming. Nubpl was amplified by PCR using forward primer CTACCACCGCAGTGAACCTT and reverse primer CAAACAAGTGGCGCAGTCTC. PCR product was column purified and Sanger sequenced.

### Animal care and approvals

This study was approved by the University of California Irvine Office of Research Institutional Animal Care & Use Committee (IACUC), Protocol #AUP19-075 and Stanford University School of Medicine IACUC Protocol #31890. Mice were housed on a standard 12 h of light-dark cycle and ad libitum food and water.* Nubpl*^*L104P+/-*^ and C57BL/6J mice were used for immunohistochemistry and immunofluorescence

### Tissue harvest and treatment

Animals were transcardially perfused with 50 ml of PBS (4 °C). Brains were harvested and embedded in optimal cutting temperature media (OCT, Sakura, 4583) for sectioning and sectioned 15 µm-thick tissue sections on a cryostat (Thermo Scientific, Model HM525NX) and placed the sections onto Fisherbrand Superfrost Plus slides (Thermo Scientific, 12-550-15).

### Fluorescent immunohistochemistry

Immunostaining was performed in brains from male mice with* Nubpl*^*L104P+/-*^ and C57BL/6J genotypes. 15 um-thick coronal sections were fixed in 10% neutral buffered formalin (Sigma-Aldrich, MKLK5486), washed with phosphate buffer saline (PBS), then permeabilized and blocked with 0.3% Triton X-100 (Sigma Aldrich, MKBF3357B), 3% bovine serum albumin (BSA) and 5% normal goat serum (NGS) in PBS for 30 minutes at room temperature. Then sections were incubated for 3 hours at room temperature with the following primary antibodies; rabbit anti-alpha-synuclein antibody (Cell Signaling, clone: D37A6, 4179BF) diluted at 1:200 or anti-nubpl antibody (Abcam, clone: EPR11833, ab171741) diluted at 1:500. After incubation with the primary antibody, sections were washed three times with PBS and incubated for 2 hours at room temperature with the following secondary antibodies: Alexa Fluor goat anti-mouse 647 (Invitrogen, A32728) and Alexa Fluor 647 anti-rabbit (Invitrogen, A27040). Nuclear counterstain was performed using a 1:10,000 Hoechst solution in PBS for five minutes. Finally, sections were mounted using ProLong Gold Antifade Mountant reagent (Invitrogen, P36930) and imaged using an ImageExpress Pico epifluorescent microscope (Molecular Devices).

### Chromogenic immunohistochemistry

Oxidative stress markers oxoguanine and nitrotyrosine were labeled in striatal and mesencephalic 15 um-thick coronal sections. After fixation in 10% neutral buffered formalin, sections were washed with PBS, endogenous peroxidase activity was blocked with 3% hydrogen peroxide (H_2_O_2_) for 15 min in PBS followed by two distilled water wash steps and a final PBS wash step. Tissue blocking was performed with 5% BSA in PBS for 30 minutes.

Primary antibodies for goat anti-oxoguanine (8-Ohdg) (dilution of 1:500) or rabbit anti nitrotyrosine antibody (dilution of 1:500) were incubated overnight at 4 °C. VisuCyte horseradish peroxidase (HRP) polymers (R&D systems, VC003 or VC004) were used to tag and were revealed with an Impact 3, 3′-diaminobenzidine (DAB) substrate kit peroxidase (Vectamount, SK-4105). Sections were dehydrated and cleared with ethanol and xylene followed by mounting with permanent mounting media (Vectamount, H5008). No hematoxylin and eosin (H&E) counterstain was performed.

## Results

**Fig. 1:**
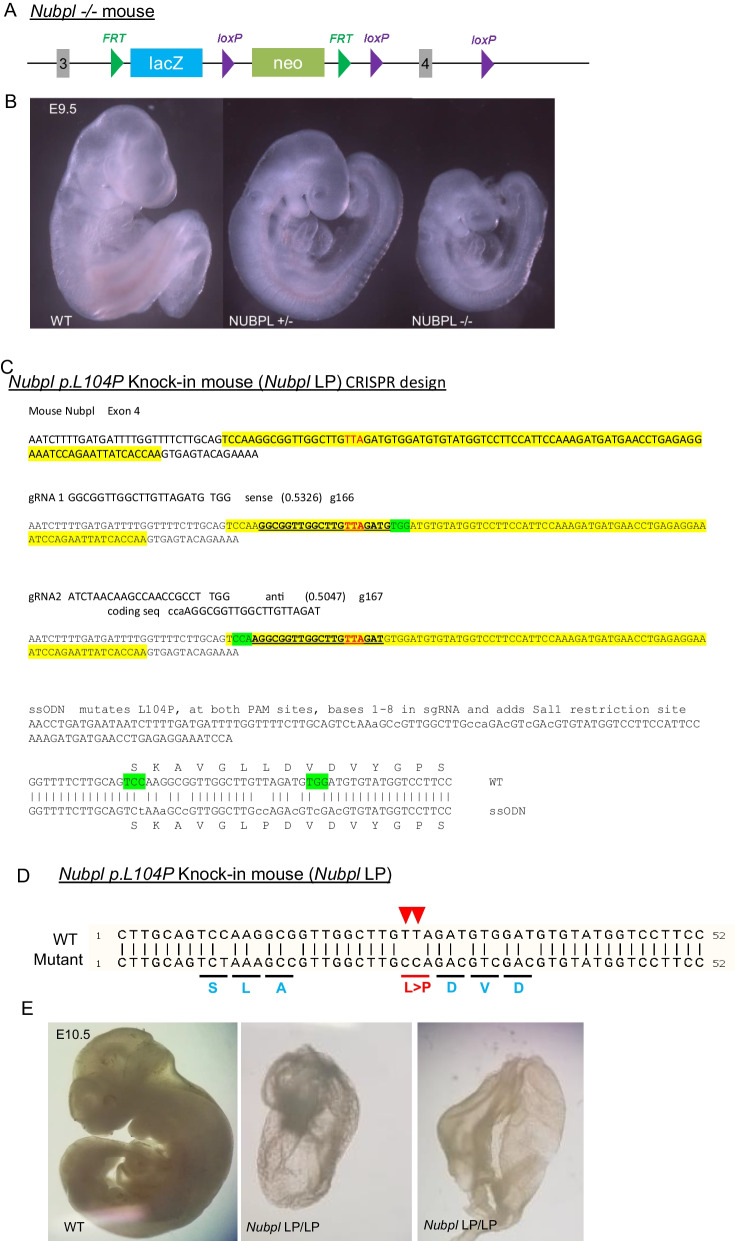
Generation of *Nubpl*^*104P+/-*^ knockout* Nubpl* ^*-/-*^ mice indicated in figure as* Nubpl* ^*-/-*^ and *Nubpl* p. L104P knock-in mice. **A** Schematic showing *Nubpl*^*tm1a*^knockout allele. **B** *Nubpl* homozygous knockout mice were sub-viable at E9.5. **C*** Nubpl p. L104P* CRISPR design. Additional silent mutations added to inhibit Cas9 cutting of p.* L104P* mutation. **D** Sequences of *Nubpl*^*104P+/-*^ heterozygous mouse for the p. L104P (TT>CC) variant. Multiple silent variants were introduced to reduce recurrent CRISPR/Cas9 targeting. **E** *Nubpl L104P/L104P* homozygous (designated as NUBPL LP/LP) mice were embryonically lethal

**Fig. 2:**
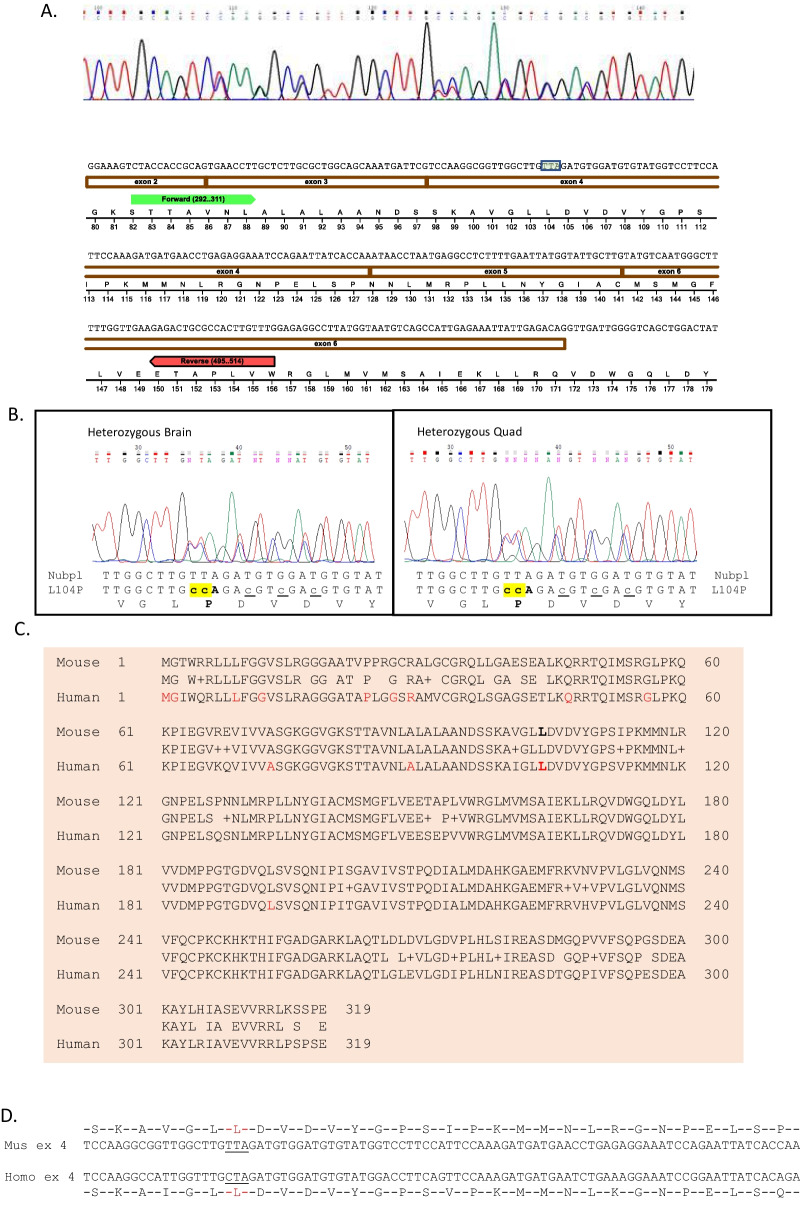
Expression of Nubpl* p. L104P* allele by cDNA sequencing. **A** Relatively similar levels of wild type and* p. L104P* is expressed. **B** PCR primers span multiple exons and exon splice junctions were correctly confirmed. Silent mutations downstream of* p. L104P* mutation were added to inhibit cutting by Cas9 Crispr during mouse model development. **C**, **D** Nubpl mouse: human amino acid homology. Known disease associated aa mutations are shown in red

Previous studies have suggested that the *Nubpl* global knockout mouse is mid-gestationally lethal, partially due to defects in placenta development. To understand the role of *Nubpl* variants in disease pathogenesis and mouse development, we generated a knock-in mouse containing patient-specific variant *Nubpl p. L104P* using CRISPR/Cas9 technology (Figs. 1, 2). The NUBPL L104 amino acid residue is conserved between human and mouse. Sanger sequencing results indicated successful knock-in of* Nubpl p.L104P* allele (Fig. 2). To confirm the expression of mutant allele, we harvested total mRNA from mouse brain cortex and performed reverse transcription to synthesize cDNA. We performed PCR to obtain the amplicon that contains the* p. L104P* mutation, followed by Sanger sequencing. Both wildtype and mutant mRNA are expressed. Furthermore, we obtained the *Nubpl* global knockout mouse from The European Mutant Mouse Archive (EMMA) (Fig. 1). Consistent with the prior study [4], we found early embryonic lethality in both models. In particular, *Nubpl* knockout mice were sub-viable at E9.5; only 3 knockout embryos were obtained out of 43 embryos. *Nubpl* ^*L104P/-*^ was born with expected Mendelian ratio. No embryos were obtained for *Nubpl* ^*L104P/L104P*^ at E10.5 (Fig. 1D, E). These data indicate that the *NUBPL p. L104P* variant behaves similar to a null allele.

**Fig. 3:**
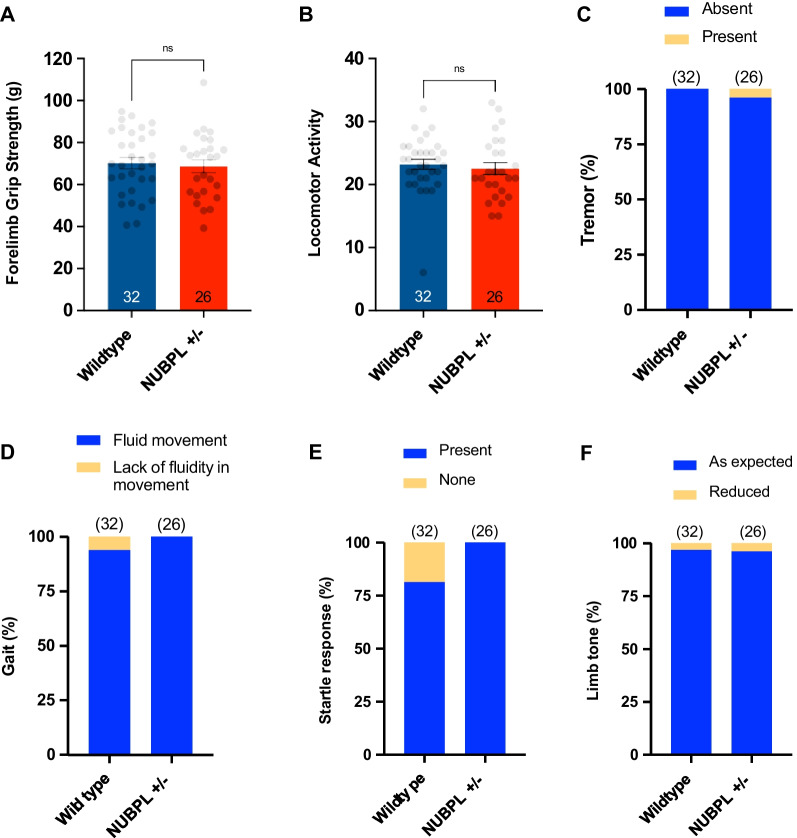
Behavioral characterization of the aging *Nubpl* heterozygous knockout mice.* Nubpl* ^*L104P+/-*^ mice indicated in figure as *Nubpl*+/− display no abnormalities in **A** forelimb grip strength, **B** locomotor activity, **C** tremor, **D** gait, **E** startle response, and **F** limb tone compared with age-matched wildtype controls

Additionally, previous studies have shown that embryonic placental defects correlate strongly with abnormal brain development [10]. In particular, genetic knockout mice with placenta defects tend to display abnormal forebrain morphology later on [10]. Interestingly, patients with *NUBPL*-associated mitochondria complex I deficiency disorders also manifest severe neurodevelopmental abnormalities including cognitive deficit and cerebellar atrophy. Recently, a patient case report revealed that haploinsufficency resulting from having one allele of *NUBPL* may increase the susceptibility of developing Parkinson’s disease (PD) in adulthood [11]. Therefore, we examined PD-associated behaviors in aging *Nubpl* hemizygous mice to further investigate the link between NUBPL deficiency and PD. At 14-month old, the *Nubpl* hemizygous mice were subject to a battery of aging phenotype screens with a high-throughput phenotyping pipeline with automated phenotype-detection strategy and visualization [12]. These tests cover a wide spectrum of behavioral and phenotypic analyses, including motor behaviors, aging parameters, vision, and emotionality tests (Fig. 3; Table 1). The *Nubpl* hemizygous mice revealed no significant abnormalities compared to the age and gender-matched wild-type mice. In particular, *Nubpl* hemizygous mice did not display reduced functional performance and feet clasping.

**Fig. 4:**
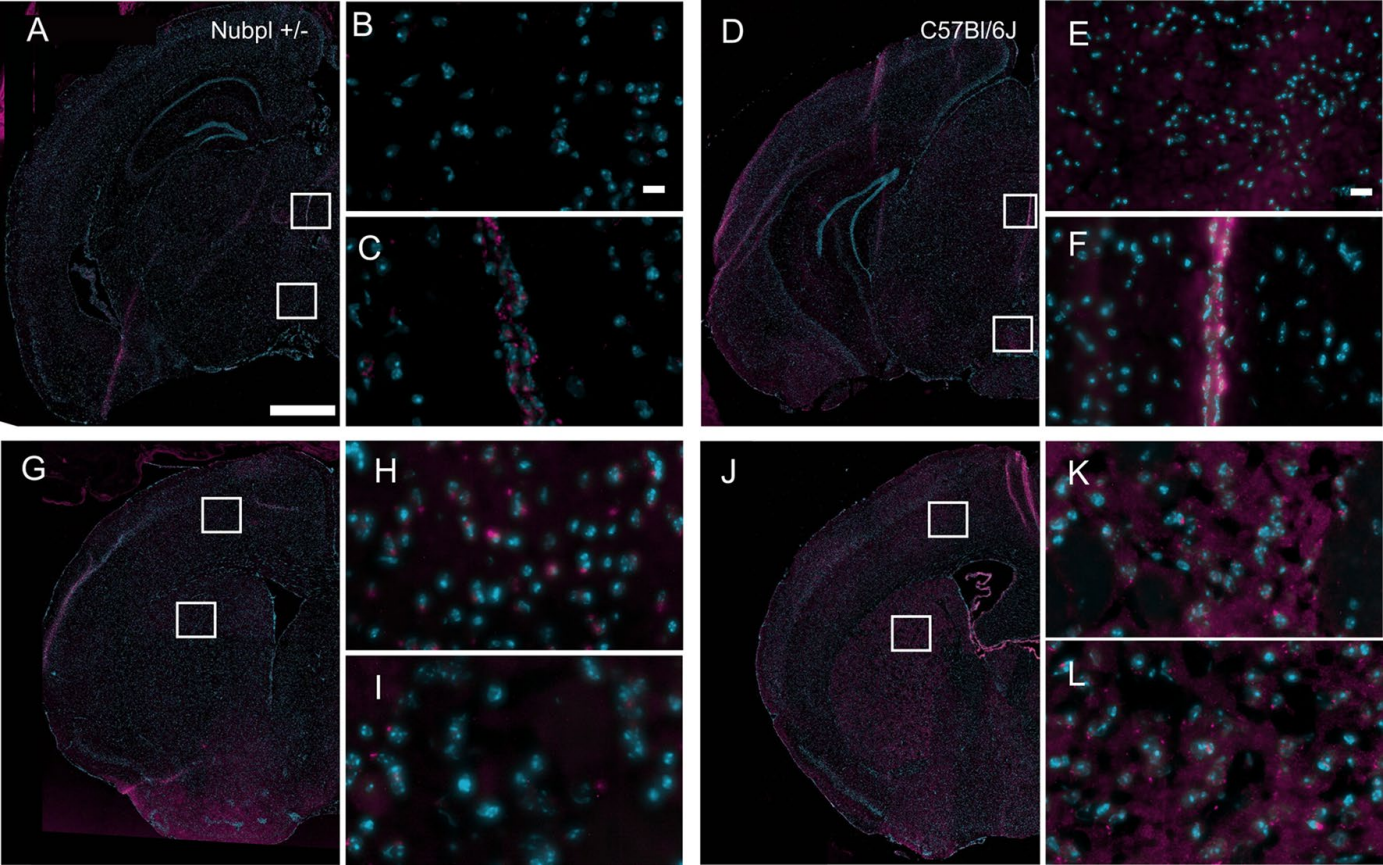
Nubpl immunofluorescence in* Nubpl* ^*L104P+/-*^ mice indicated in figure as Nubpl+/− and C57BL/6J brains. **A**, **D**, **G**, **J** Panoramic hemi-brain; Nubpl expression in magenta and nuclear counterstain (Hoechst) in blue. Approximate coordinate -3 anteroposterior (AP) related to Bregma; **A**–**C** Mesencephalic coronal section of a* Nubpl*^*L104P+/-*^ male; **G**–**I** Striatal section of Nubpl +/- male; D-F. Mesencephalic coronal section; **J**–**L** Striatal coronal section of a 10 months old C57BL/6J control male; **C**, **F** magnification of the third ventricle; **B**, **E** Zoom into the interpeduncular nucleus of the mesencephalon; **H** **K** Magnification in the caudoputamen; **I**, **L** Magnification in the cortex. Scale bar for **A**, **G** represents 500 µm; **B**, **E**, represents 10 µm

To characterize the pathological changes in *Nubpl* hemizygous mice, Nubpl protein was analyzed by immunofluorescence. In the *Nubpl* hemizygous mice, we found an overall decreased expression of Nubpl in both the striatal and mesencephalic sections compared to the C57BL/6J strain (Fig. 4). Histological analysis of the PD-related protein alpha-synuclein was performed to characterize the expression pattern in the two main brain regions affected in PD: striatum and substantia nigra. This analysis did not show any changes in alpha-synuclein expression compared to a 10-month-old C57BL/6J mouse (Fig. 7), which supports the behavioral and phenotypic analysis.

**Fig. 5:**
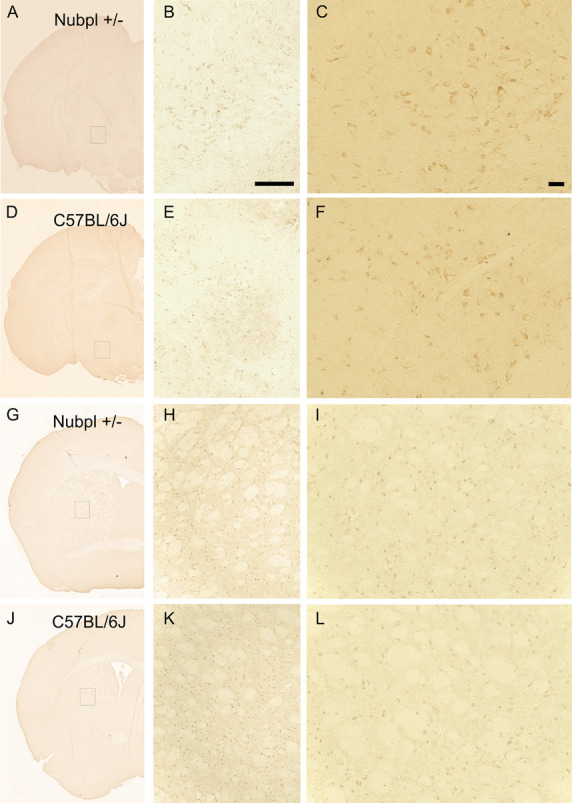
NTT (nitrotyrosine) expression in *Nubpl*^*L104P/-*^mice indicated in figure as Nubpl +/− and C57BL/6J brains. **A**–**C**, **G**–**I** Panoramic 3,3′-Diaminobenzidine (DAB) hemi-brain staining of Nubpl+/− male; **D**–**F**, **J**–**L** Hemi-brain stain of a 10-month old C57BL/6J control male; **B**, **E** Zoom in the third ventricle; **C**, **F** Magnification of the third ventricle; **H**, **K** Zoom in the caudoputamen-lateral ventricle; **I**, **L** Magnification of the caudoputamen-lateral ventricle. Scale bar for **B** represents 250 µm, for C represents 100 µm

**Fig. 6:**
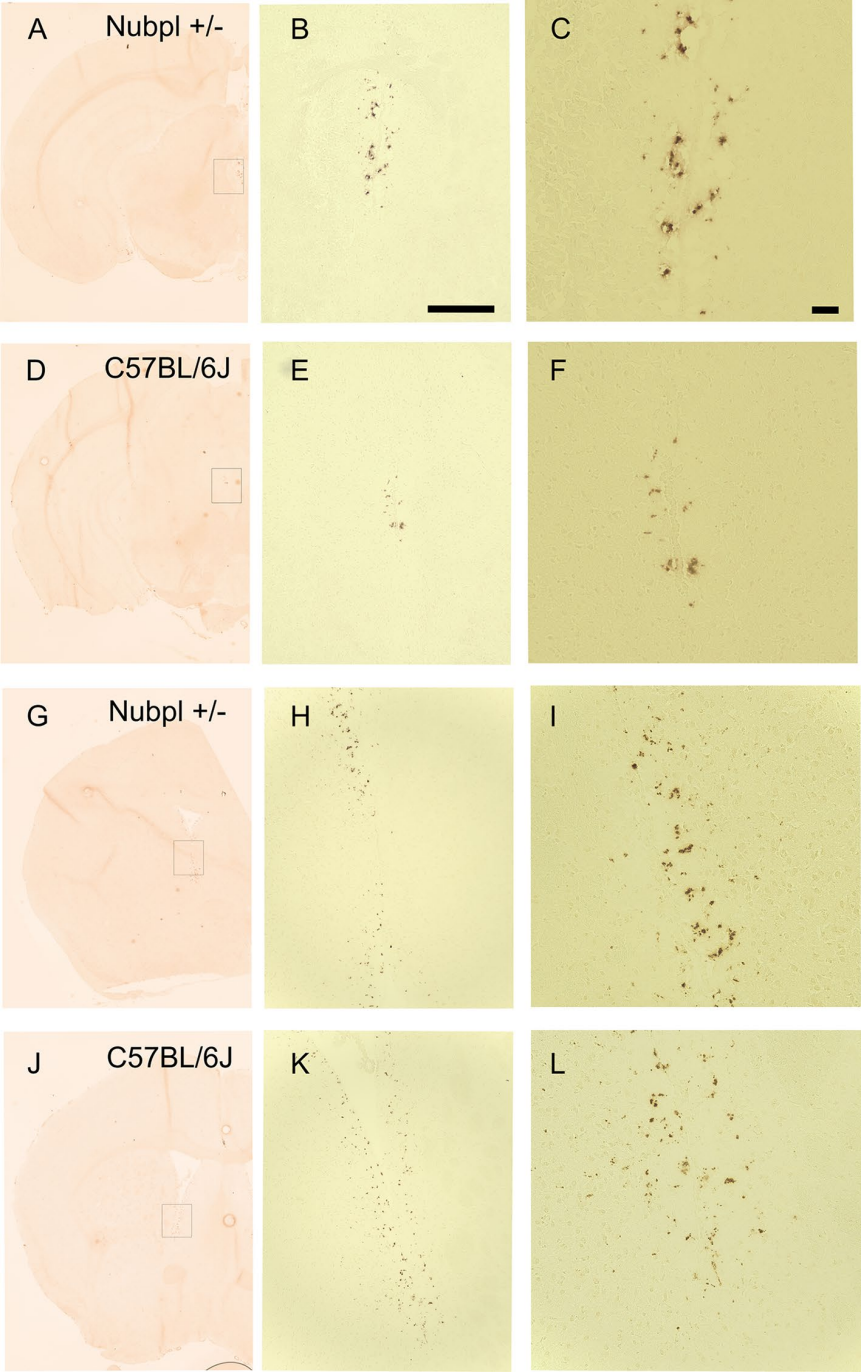
8-Oxoguanine (8-OHDG) expression in* Nubpl* ^*L104P/-*^ mice indicated in figure as Nubpl +/− and C57BL/6J brains. **A**–**C**, **G**–**I** Panoramic 3,3′-Diaminobenzidine (DAB) hemi-brain staining of Nubpl+/− male; **D**–**F**, **J**–**L** Hemi-brain stain of a 10-month old C57BL/6J control male; **B**, **E** Zoom in the substantia nigra (SN); **C**, **F** Magnification of the SN; **H**, **K** Zoom in the caudoputamen. **I**, **L** Magnification of the caudoputamen. Scale bar **B** scale bar: 250 µm, **C** 100 µm

NUBPL is a Fe/S protein that plays a critical role in the assembly of the respiratory complex I, part of the respiratory chain in the mitochondria [13, 14]. Depletion or impairment of the correct assembly of the respiratory chain could cause an increase in reactive oxygen species leading to an increase in oxidative stress [15, 16]. Therefore, we also evaluated nitrotyrosine (NTT) and oxoguanine (8-Ohdg) with immunohistochemistry. There were no evident changes in the expression patterns of these two oxidative stress markers (Figs. 5, 6).

**Fig. 7:**
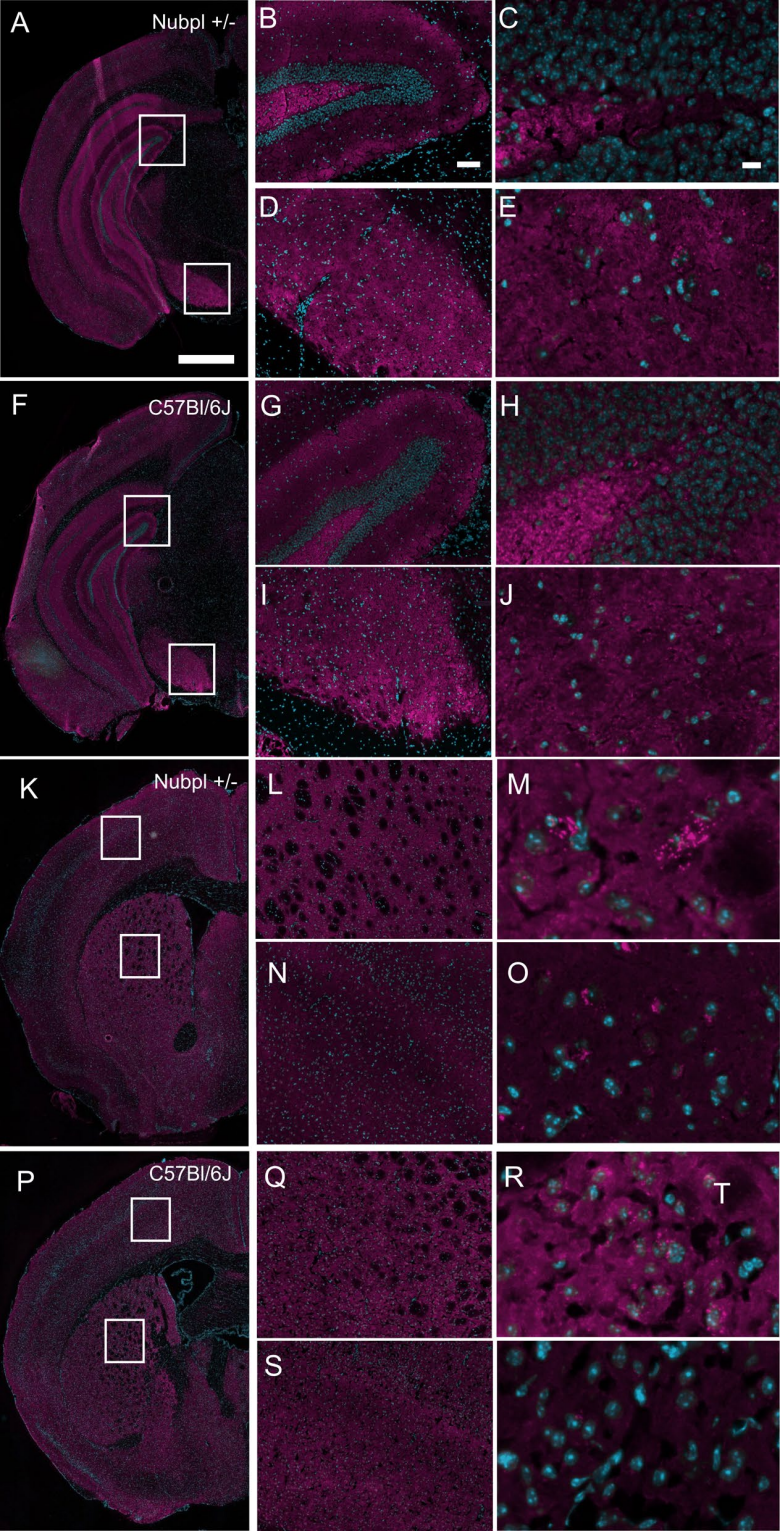
Alpha-synuclein expression in* Nubpl*^*L104P/-*^ mice indicated in figure as Nubpl+/− and C57BL/6J brains. **A**, **F**, **K**, **P** Panoramic hemi-brain; alpha-synuclein in magenta and nuclear counterstain (Hoechst) in blue. Approximate AP coordinate -3 related to Bregma; A-E. Mesencephalic coronal section of a Nubpl+/− male, **K**–**O** Striatal section of Nubpl+/− male; **F**–**J** Mesencephalic coronal section; **P**–**T** Striatal coronal section of a 10-month-old C57BL/6J control male; **B**, **G** Zoom into the hippocampal formation including the dental gyrus granule cell layer, polymorph layer, and molecular layer; **C**, **H** Magnification of the dentate gyrus, polymorph layer; **D**, **I** Zoom into the substantia nigra, reticular part, and part of the cerebal peduncle; **E**, **J** Magnification of the substantia nigra, reticular part; **L**, **Q** Zoom in the caudoputamen; **M**, **R** Magnification in the caudoputamen; **N**, **S** Zoom into the cortex; **O**, **T** Magnification in the cortex. Scale bar for **A**, **F** represents 500 µm, **B**, **D**, **G**, **I** represents 100 µm, **C**, **E**, **H**, **J** represents 10 µm

## Discussion

Our studies have provided insights into the pathogenicity of the *NUBPL* p. L104P variant. Patients harboring the *NUBPL* p. L104P variant display severe cerebellar degeneration along with intellectual disability and global developmental delay [3]. Previous studies characterizing the yeast mimics of NUBPL p. L104P mutation suggest that this amino acid change likely results in misfolding of the NUBPL protein. We found that the *Nubpl* p. L104P mouse was lethal at the mid-gestational stage, similar to the *Nubpl*-null mouse, thus it is likely that the p. L104P variant is a hypomorph that mimics the null allele. Thus, a tissue-specific knockout model of *Nubpl* might be a more advantageous model to help delineate the function of NUBPL and pursue therapeutic strategies for complex I deficiency disorders.

Despite our intention to determine the complex I activity in the NUBPL p. L104P homozygous mice, it is difficult because embryonic lethality happens too early in the developmental stage to obtain enough tissue to perform both genotyping experiments and complex I activity assay for each embryo. For the same reason, we could not perform histological analysis for the mutant embryos. Moreover, establishing cell lines including mouse embryonic fibroblast is challenging with normally developed embryos from E9-E10, let alone defective embryos due to NUBPL deficiency.

Even though we did not observe significant behavioral abnormalities in aging *Nubpl* heterozygous mice, we cannot exclude the possibility that partial loss of function leads to increased susceptibility to PD. It is plausible that our end-point behavioral analysis was not sensitive enough to detect abnormalities or PD-associated behaviors, or mice were not sufficiently aged to detect one. At 14 months, we also did not detect frank neuropathology related to oxidative stress or accumulation of alpha-synuclein. Older mice may manifest late-onset PD-like features. In future analysis, a brain-specific knockout model may also provide more insights into how NUBPL plays a role in the central nervous system.

Altogether, our attempt to generate a mouse model of *NUBPL*-associated mitochondrial deficiency has suggested that strategies of global knock-in with *Nubpl p. L104P* and *Nubpl* knockout are deleterious to embryogenesis, and future studies will be critical to understanding and bypass the role of NUBPL in early development. Generation of tissue-specific *Nubpl* knockout or knock-in mice that harbor less deleterious variants to understand disease pathogenesis is necessary to help in developing models to study potential treatments for this rare severe disorder.

## Data Availability

Please contact author for data requests.
